# Polymorphisms in mitotic checkpoint-related genes can influence survival outcomes of early-stage non-small cell lung cancer

**DOI:** 10.18632/oncotarget.18693

**Published:** 2017-06-27

**Authors:** Hyo Gyoung Kang, Seung Soo Yoo, Jin Eun Choi, Mi Jeong Hong, Sook Kyung Do, Cheng Cheng Jin, Soyoun Kim, Won Kee Lee, Sun Ha Choi, So Yeon Lee, Hyun Jung Kim, Shin Yup Lee, Jaehee Lee, Seung Ick Cha, Chang Ho Kim, Yangki Seok, Eungbae Lee, Sukki Cho, Sanghoon Jheon, Jae Yong Park

**Affiliations:** ^1^ Department of Biochemistry and Cell Biology, School of Medicine, Kyungpook National University, Daegu, Republic of Korea; ^2^ Department of Cell and Matrix Research Institute, School of Medicine, Kyungpook National University, Daegu, Republic of Korea; ^3^ Department of Internal Medicine, School of Medicine, Kyungpook National University, Daegu, Republic of Korea; ^4^ BK21 Plus KNU Biomedical Convergence Program, Department of Biomedical Science, Kyungpook National University, Daegu, Republic of Korea; ^5^ Department of Preventive Medicine, School of Medicine, Kyungpook National University, Daegu, Republic of Korea; ^6^ Department of Thoracic Surgery, School of Medicine, Kyungpook National University, Daegu, Republic of Korea; ^7^ Department of Thoracic and Cardiovascular Surgery, School of Medicine, Seoul National University, Seoul, Republic of Korea

**Keywords:** polymorphisms, mitosis, mitotic checkpoint, survival outcome, lung cancer

## Abstract

This study was conducted to investigate the association between variants in mitotic checkpoint-related genes and clinical outcomes of non-small cell lung cancer (NSCLC). A total of 766 patients with NSCLC who underwent curative surgery were enrolled. Among the 73 variants evaluated, 4 variants were related with survival outcomes. *BUB3* rs7897156C>T was associated with worse overall survival under a recessive model (adjusted hazard ratio = 1.58, 95% confidence interval = 1.07–2.33, *P* = 0.02). *AURKB* rs1059476G>A was associated with better overall survival under a recessive model (adjusted hazard ratio = 0.64, 95% confidence interval = 0.41–0.99, *P* = 0.05). *PTTG1* rs1895320T>C and *RAD21* rs1374297C>G were associated with worse disease-free survival. In the functional study, relative luciferase activity was higher at the *BUB3* rs7897156T allele compared to that at the C allele. Western blot showed that the phosphorylation of AKT and mTOR in the *AURKB* variant-type (M^298^) was significantly lower than in the *AURKB* wild-type (T^298^). We found that 4 variants of mitotic checkpoint-related genes were associated with survival outcomes in patients with surgically resected NSCLC. Particularly, our results suggest that *BUB3* rs7897156C>T and *AURKB* rs1059476G>A are functional variants.

## INTRODUCTION

Accurate segregation of chromosomes during mitosis is important to the survival of human cells. Errors in mitosis result in cells with an abnormal number of chromosomes, known as aneuploidy, which can lead to cell-cycle arrest, cell death, or tumorigenesis [1, 2]. Whether aneuploidy is a cause or consequence of cancer remains controversial; aneuploidy has been detected in most solid tumors, including lung cancer [2-4]. Chromosome instability, defined as a high rate of either gain or loss of whole chromosomes and causing aneuploidy, is also one of the hallmarks of human solid tumors [2, 5].

During mitosis, there are various checkpoints to ensure the fidelity of chromosome segregation. Checkpoint with FHA and RING finger domains (CHFR) and spindle assembly are examples of mitotic checkpoints [6, 7]. A number of genes, such as mitotic arrest deficient (MAD) 1–3 and budding uninhibited by benzimidazole (BUB) 1–3, are also related to mitotic checkpoints to guarantee accurate chromosome segregation [8-11]. Defects in mitotic checkpoints can cause chromosome instability, which contributes to tumorigenesis. [12, 13].

Mutations in mitotic checkpoint-related genes such as *BUB1* and *MAD1L1* have been reported in various human cancers, including lung cancer [14-17]. We hypothesized that mutations in mitotic checkpoint-related genes affect not only tumorigenesis, but also the clinical outcomes of cancers. Therefore, we evaluated the association between potentially functional single nucleotide polymorphisms (SNPs) in mitotic checkpoint-related genes and the clinical outcomes of early-stage of non-small cell lung cancers (NSCLC).

## RESULTS

### Patient characteristics and clinical predictors

The clinical and pathological characteristics of patients and their association with overall survival (OS) and disease-free survival (DFS) are shown in Table 1. Pathological stage was significantly associated with both OS and DFS in univariate analysis (log-rank *P* = 9 × 10^-13^ and 4 × 10^-18^, respectively). Age, gender, and smoking status were also associated with OS (log-rank *P* = 0.01, 7 × 10^-4^ and 8 × 10^-4^, respectively).

**Table 1:** Univariate analysis for overall survival and disease-free survival by clinicopathologic features

Variables		Overall survival				Disease-free survival		
	No. of cases	No. of death (%)^a^	5Y-OSR (%)^b^	Log-Rank *P*	No. of event (%)^a^	5Y-DFSR (%)^b^	Log-Rank *P*
Overall	766		214 (27.9)	61		337 (44.0)	44	
Age (years)								
< 65	375		94 (25.1)	66	0.01	160 (42.7)	48	0.14
≥ 65	391		120 (30.7)	55		177 (45.3)	40	
Gender								
Male	560		177 (31.6)	58	7 × 10^-4^	260 (46.4)	42	0.09
Female	206		37 (18.0)	71		77 (37.4)	53	
Smoking status								
Never	229		43 (18.8)	73	8 × 10^-4^	89 (38.9)	50	0.12
Ever	537		171 (31.8)	56		248 (46.2)	42	
Pack-years^c^								
< 40	250		72 (28.8)	57	0.27	111 (44.4)	42	0.79
≥ 40	287		99 (34.5)	55		137 (47.7)	42	
Histological type								
SCC	344		108 (31.4)	59	0.30	151 (43.9)	46	0.35
AC	405		100 (24.7)	63		176 (43.5)	43	
LCC	17		6 (35.3)	61		10 (58.8)	39	
Pathologic stage								
I	363		57 (15.7)	76	9 × 10^-13^	102 (28.1)	61	4 × 10^-18^
II-IIIA	403		157 (39.0)	48		235 (58.3)	30	
Adjuvant therapy^d^								
No	182		75 (41.7)	47	0.71	104 (57.8)	35	0.51
Yes	221		88 (36.8)	49		131 (58.7)	25	

SCC, squamous cell carcinoma; AC, adenocarcinoma; LCC, large cell carcinoma.

^a^Row percentage.

^b^Five year-overall survival rate (5Y-OSR) and 5 year-disease free survival rate (5Y-DFSR), proportion of survival derived from Kaplan-Meier analysis.

^c^In ever-smokers.

^d^In pathologic stage II + IIIA: 184 cases received paclitaxel- cisplatin chemotherapy, 11 cases received radiotherapy, and 26 cases received chemotherapy and radiotherapy.

### Association between SNPs and survival outcomes

**Figure 1:**
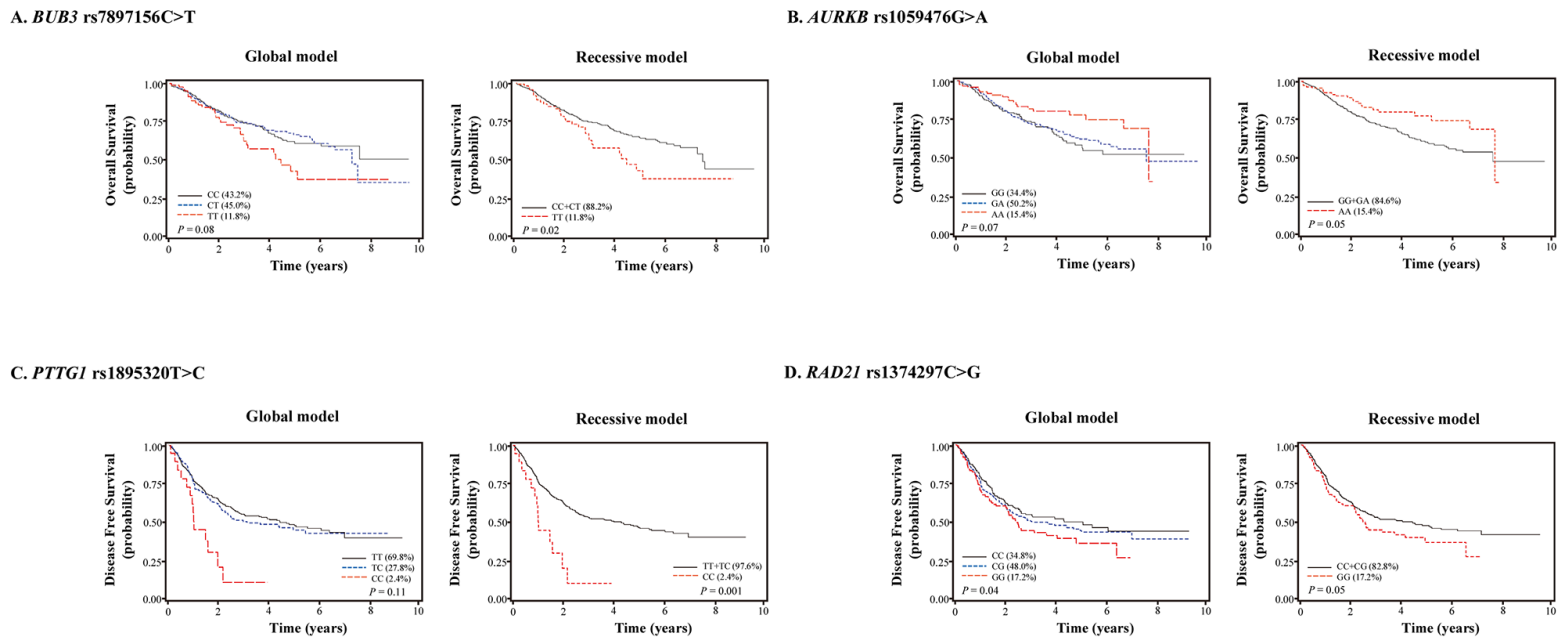
Kaplan-Meier plots of overall survival or disease-free survival according to **(A)** *BUB3* rs7897156C>T, **(B)** *AURKB* rs1059476G>A, **(C)** *PTTG1* rs1895320T>C, and **(D)** *RAD21* rs1374297C>G genotypes. *P* values, multivariate Cox proportional hazard model.

The 73 SNPs evaluated and results of multivariate analyses are shown in Supplementary Table 1. Among the 73 SNPs examined, 4 SNPs (rs7897156, rs1059476, rs1895320, and rs1374297) were associated with survival outcomes. *BUB3* rs7897156C>T was associated with worse overall survival under a recessive model (CC+CT (88.2%) *vs.* TT (11.8%), adjusted hazard ratio [aHR] = 1.58, 95% confidence interval [CI] = 1.07–2.33, *P* = 0.02; Table 2 and Figure 1). Aurora kinase B (AURKB) rs1059476G>A was associated with better overall survival under a recessive model (GG+GA (84.6%) *vs.* AA (15.4%), aHR = 0.64, 95% CI = 0.41–0.99, *P* = 0.05; Table 2 and Figure 1). Although significant *P* values were not reached, the same trends as OS were observed in DFS for *BUB3* rs7897156 and *AURKB* rs1059476 (Table 2). Pituitary tumor-transforming 1 (PTTG1) rs1895320 and RAD21 cohesin complex component (RAD21) rs1374297 were associated with worse DFS (under a recessive model, TT+TC (97.6%) *vs.* CC (2.4%), aHR = 2.46, 95% CI = 1.43–4.23, *P* = 0.001 and under a codominant model, CC (34.8%) *vs.* CG (48.0%) *vs.* GG (17.2%), aHR = 1.18, 95% CI = 1.01–1.38, *P* = 0.04, respectively; Table 2 and Figure 1). There was no association between 4 SNPs and EGFR, ALK, and RET (Supplementary Table 2).

**Table 2:** Overall survival and disease-free survival according to genotypes in patients with non-small lung cancer

Gene/SNP			Overall survival					Disease-free survival				
	Genotype^e^	No. of cases (%)^a^	No. of deaths (%)^b^	5Y-OSR (%)^c^	Log-rank *P*	HR (95% CI)^c^	*P*^d^	No. of events (%)^b^	5Y-DFSR (%)^c^	Log-rank *P*	HR (95% CI)^c^	*P*^d^
*BUB3*	CC	325 (43.2)	88 (27.1)	61	0.08	1.00		144 (44.3)	42	0.57	1.00	
rs7897156	CT	339 (45.0)	90 (26.6)	67		1.04 (0.77-1.39)	0.81	144 (42.5)	50		0.97 (0.77-1.22)	0.77
	TT	89 (11.8)	31 (34.8)	38		1.61 (1.06-2.44)	0.03	39 (43.8)	34		1.25 (0.87-1.79)	0.23
	Dominant				0.54	1.14 (0.86-1.50)	0.36			0.71	1.01 (0.81-1.26)	0.90
	Recessive				0.02	1.58 (1.07-2.33)	0.02			0.40	1.27 (0.91-1.78)	0.17
	Codominant					1.20 (0.98-1.47)	0.08				1.07 (0.90-1.26)	0.47
*AURKB*	GG	259 (34.4)	75 (29.0)	54	0.07	1.00		118 (45.6)	40	0.09	1.00	
rs1059476	GA	378 (50.2)	109 (28.8)	62		0.93 (0.69-1.25)	0.62	169 (44.7)	45		0.89 (0.70-1.12)	0.32
	AA	116 (15.4)	22 (19.0)	75		0.61 (0.38-0.98)	0.04	40 (34.5)	55		0.71 (0.50-1.02)	0.06
	Dominant				0.21	0.85 (0.64-1.13)	0.27			0.15	0.85 (0.67-1.06)	0.15
	Recessive				0.03	0.64 (0.41-0.99)	0.05			0.05	0.76 (0.55-1.07)	0.11
	Codominant					0.82 (0.67-1.01)	0.07				0.86 (0.73-1.01)	0.06
*PTTG1*	TT	520 (69.8)	148 (28.5)	60	0.05	1.00		214 (41.2)	47	0.001	1.00	
rs1895320	TC	207 (27.8)	49 (23.7)	67		0.74 (0.53-1.03)	0.07	93 (44.9)	44		1.01 (0.79-1.29)	0.94
	CC	18 (2.4)	9 (50.0)	48		1.52 (0.77-2.99)	0.23	14 (77.8)	10		2.47 (1.42-4.27)	0.001
	Dominant				0.31	0.80 (0.59-1.09)	0.16			0.17	1.10 (0.87-1.39)	0.45
	Recessive				0.05	1.65 (0.84-3.23)	0.14			0.0001	2.46 (1.43-4.23)	0.001
	Codominant					0.90 (0.69-1.18)	0.44				1.18 (0.96-1.46)	0.11
*RAD21*	CC	260 (34.8)	69 (26.5)	62	0.59	1.00		104 (40.0)	50	0.18	1.00	
rs1374297	CG	358 (48.0)	99 (27.7)	62		1.06 (0.78-1.45)	0.70	157 (43.9)	44		1.12 (0.87-1.43)	0.39
	GG	128 (17.2)	38 (29.7)	57		1.26 (0.85-1.88)	0.26	62 (48.4)	37		1.41 (1.03-1.94)	0.03
	Dominant				0.59	1.11 (0.83-1.49)	0.48			0.18	1.19 (0.94-1.50)	0.16
	Recessive				0.32	1.22 (0.85-1.73)	0.28			0.10	1.32 (1.00-1.75)	0.05
	Codominant					1.11 (0.91-1.36)	0.29				1.18 (1.01-1.38)	0.04

^a^Column percentage.

^b^Row percentage.

^c^Five year-overall survival rate (5Y-OSR) and 5 year-disease free survival rate (5Y-DFSR), proportion of survival derived from Kaplan-Meier analysis.

^d^Hazard ratios (HRs), 95% confidence intervals (CIs) and corresponding *P*-values were calculated using multivariate Cox proportional hazard models, adjusted for age, sex, smoking status, tumor histology, pathologic stage and adjuvant therapy.

^e^Genotype failure: 13 cases for the rs7897156, 13 cases for the rs1059476, 21 cases for the rs1895320, and 20 cases for the rs1374297.

### Effect of rs7897156C>T on promoter activity of *BUB3*

**Figure 2:**
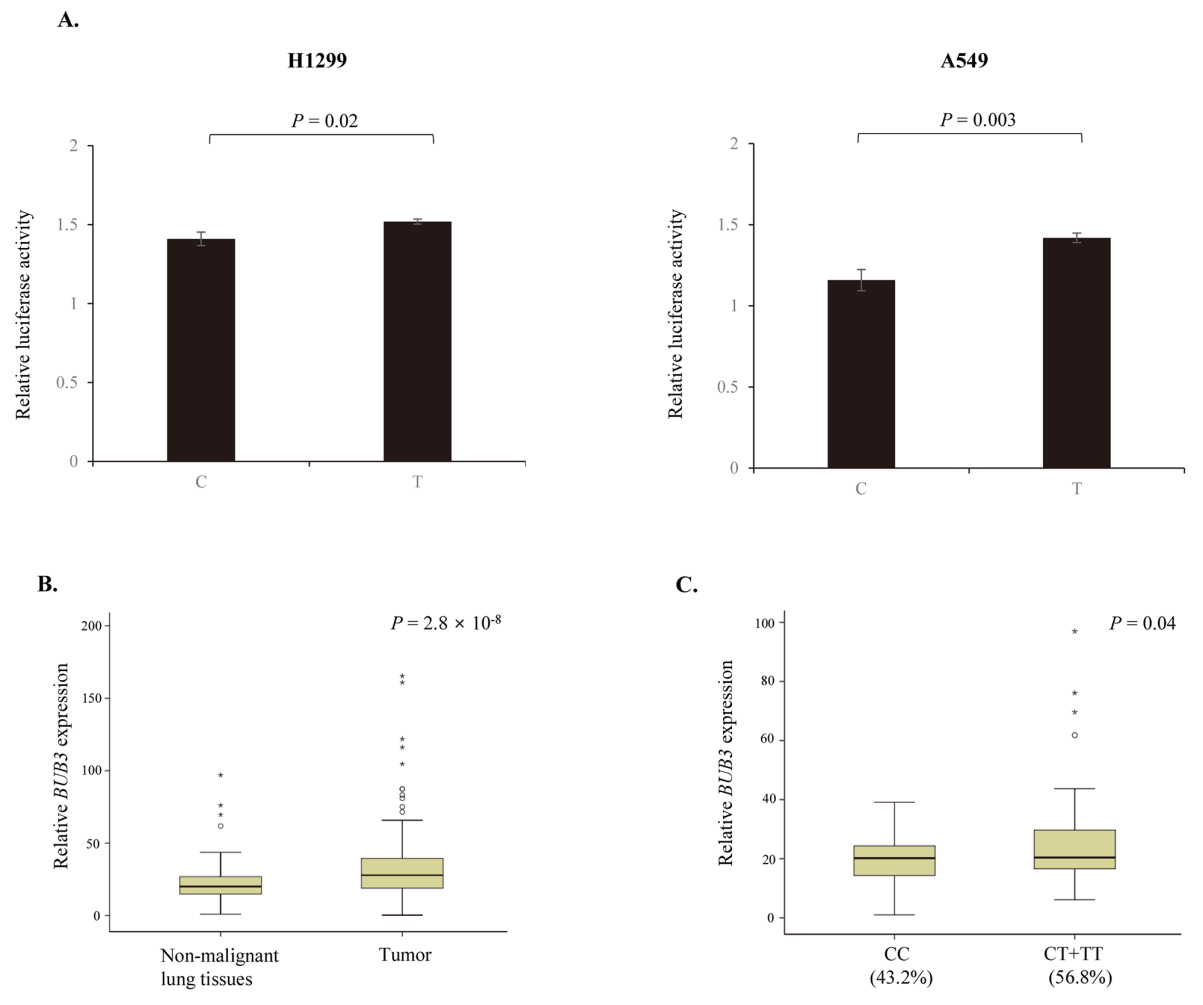
Effect of *BUB3* rs7897156C>T **(A)** Functional analysis of *BUB3* rs7897156C>T by luciferase reporter assay. Transcription activity of rs7897156C>T was measured using a Dual-Luciferase Reporter Assay System in the H1299 and A549 cell lines. Firefly luciferase activity was normalized to *Renilla* luciferase activity. **(B)** mRNA expression levels of *BUB3* in tumor and non-malignant lung tissues. **(C)** *BUB3* mRNA expression by the rs7897156C>T genotypes in non-malignant lung tissues. The *BUB3* mRNA expression levels and association with the rs7897156C>T genotypes were examined in 114 cases with tumor and paired non-malignant lung tissues. The mRNA expression level of *BUB3* was normalized to that of the β-actin gene. *P* values, Student’s *t*-test.

rs7897156C>T is located at the 5′ untranslated region of *BUB3* and can change the promoter activity of *BUB3*. We investigated the effect of rs7897156C>T on the promoter activity of *BUB3* using a luciferase assay in the H1299 and A549 NSCLC cell lines. The rs7897156T allele showed significantly higher luciferase activity of the *BUB3* promoter compared to the 7897156C allele in both cell lines (*P* = 0.02 and 0.003, respectively; Figure 2A).

### Effect of rs7897156C>T on *BUB3* mRNA expression

Increased *BUB3* expression was observed in tumor tissues compared to in paired non-malignant lung tissues (*P* = 2.8 × 10^-8^; Figure 2B). To identify the functional effect of *BUB3* rs7897156C>T, we evaluated the mRNA expression according to the rs7897156 genotypes. *BUB3* mRNA expression was higher for the rs7897156CT or TT genotypes (56.8%) than for the rs7897156CC genotype (43.2%) in non-malignant lung tissues (*P* = 0.04; Figure 2C).

### Functional prediction of *AURKB* rs1059476G>A

**Figure 3:**
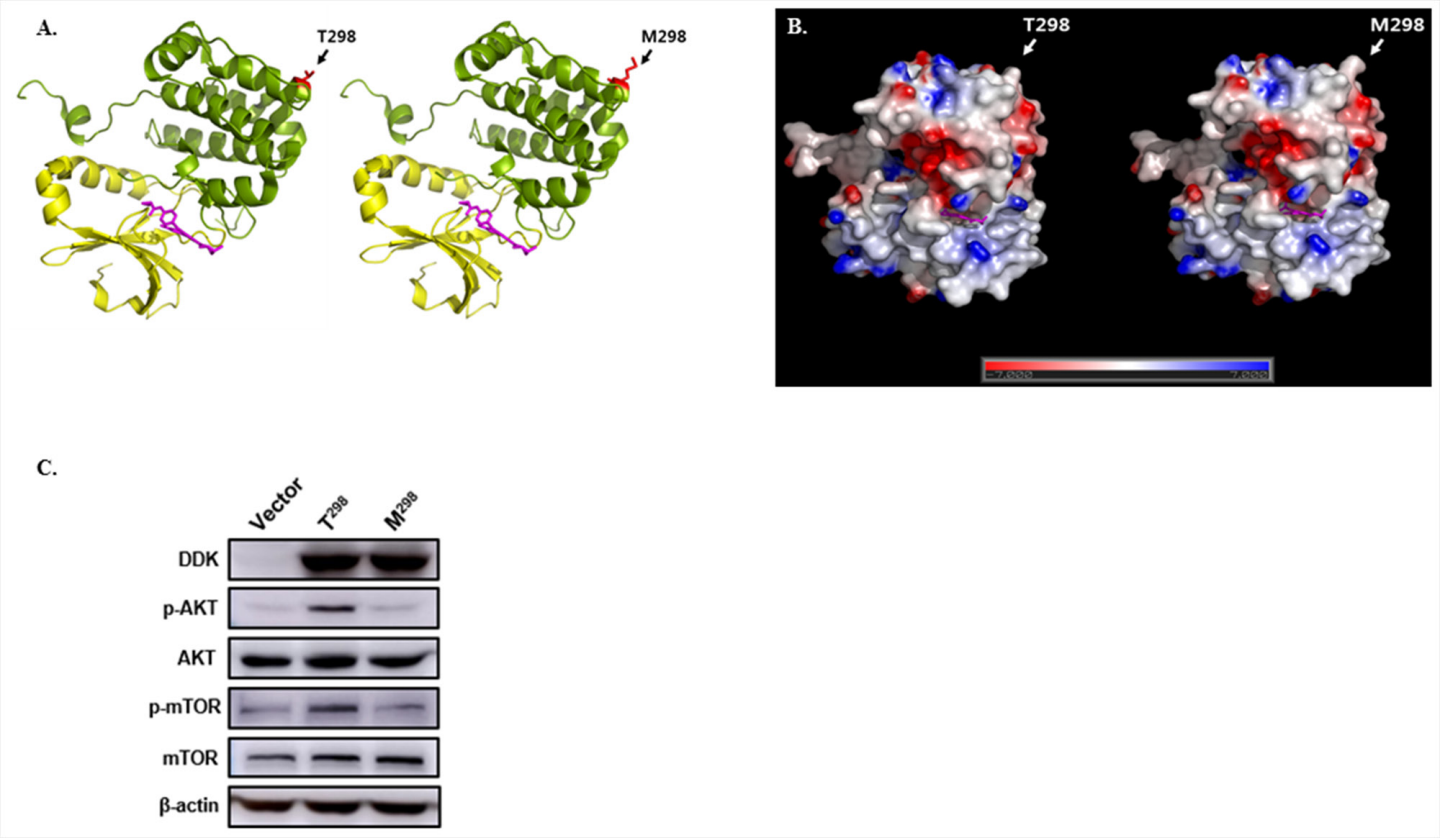
Effect of *AURKB* rs1059476G>A **(A)** Structure model of Aurora kinase (PDB ID: 4AF3) wild-type (T298) and variant type (M298). Kinase domain (yellow) and substrate selective region (green) are shown with kinase inhibitor, VX680 (magenta). Single nucleotide polymorphism residue is highlighted in red. **(B)** The surface electrostatic potential of wild-type and variant-type was calculated using DelPhi calculation; all figures were constructed using Pymol (http://pymol.org). **(C)** Western blot of DDK-tagged *AURKB* wild-type (T^298^) and variant-type (M^298^), for phosphorylated AKT (p-AKT), total AKT, phosphorylated mTOR (p-mTOR), and total mTOR, which were transfected into 293T cells. β-actin was used as internal control.

*AURKB* rs1059476G>A is a non-synonymous SNP. Changing the rs1059476G-to-A results in an amino acid change of threonine to methionine at codon 298. We evaluated whether this amino acid change affects protein function using DelPhi calculation and Pymol (http://pymol.org). As shown in Figure 3, the Thr-to-Met change at codon 298 provides increased hydrophobicity of Aurora kinases B.

### *In vitro* effect of rs1059476G>A

To assess the functional importance of *AURKB* variant-type (M^298^) identified in this study, the effect of the variant-type (M^298^) on AURKB was investigated *in vitro*. DDK-tagged wild-type (T^298^) and variant-type (M^298^) were transfected into 293T cells. Western blot for the phosphorylation of AKT and mTOR in the variant-type (M^298^) was significantly lower compared with that in the wild-type (T^298^) (Figure 3C). Total protein level of AKT and mTOR was not changed. These findings suggest that *AURKB* variant-type (M^298^) decreases the phosphorylation of AKT and mTOR.

## DISCUSSION

In the present study, we evaluated the effect of 73 SNPs in 25 mitotic-checkpoint related genes on the survival outcomes in patients with surgically resected NSCLC. We found that 4 SNPs (rs7897156, rs1059476, rs1895320, and rs1374297) were associated with OS or DFS.

BUB3 is a mitotic checkpoint protein that is essential for the establishment of correct kinetochore-microtubule attachments [18]. BUB3, in complex with BUB1, delays the onset of anaphase until all mitotic chromosomes are properly attached to the mitotic spindle [8, 19]. Overexpression of BUB3 was reported in several cancers such as breast and gastric cancer [20, 21]. In this study, *BUB3* mRNA expression was higher in lung tumor tissues than in non-malignant lung tissues. Up-regulation of mitotic checkpoint gene expression is frequently observed in tumors, but the reason remains unclear. Overexpression of mitotic checkpoint genes may occur because of compensatory mechanisms for other defects in mitotic checkpoint function. Alternatively, overexpression of mitotic checkpoint genes may result in defective mitotic checkpoint function [22].

Associations between the overexpression of mitotic checkpoint genes and poor prognosis have been reported in several cancers [23-27]. In the present study, a higher level of *BUB3* expression was observed in lung tumor, and worse OS in patients with the *BUB3* rs7897156TT genotype was observed. Our finding is biologically plausible given the putative function of the SNP. In the *in vitro* luciferase assay, the rs7897156C-to-T change increased the activity of the *BUB3* promoter. In addition, the rs7897156CT or TT genotypes showed significantly higher *BUB3* mRNA expression than the rs7897156CC genotype. These results suggest that *BUB3* rs7897156C>T is a functional SNP. Therefore, the rs7897156C-to-T change may have resulted in overexpression of *BUB3* and may affect mitotic checkpoint function to influence the prognosis of patients with NSCLC. To the best of our knowledge, this is the first study to show that *BUB3* rs7897156C>T is a functional SNP and can affect prognosis of NSCLC.

The family of Aurora kinases (A, B, and C) act as key regulators of mitotic cell division by regulating the functions of centrosomes, bipolar spindle assembly, and chromosome segregation [28]. Up-regulation of Aurora kinases has been reported in various human cancers, and therapeutic inhibition of these kinases are actively researched as anti-mitotic therapies for cancers [9, 28]. In this study, patients with the *AURKB* rs1059476AA genotype showed better OS than those with the *AURKB* rs1059476GA or GG genotypes. The rs1059476G>A is located in exon of *AURKB* and a non-synonymous SNP. The rs1059476G-to-A change results in the replacement of threonine to methionine at codon 298. The prediction of the three-dimensional model using DelPhi calculation and Pymol program showed increased hydrophobicity of Aurora kinases B by the amino acid change of T298M. Western blot for the phosphorylation of AKT and mTOR was decreased in *AURKB* the variant-type (M^298^) compared to *AURKB* the wild-type (T^298^). However, the biological mechanism between the *AURKB* rs1059476G>A and survival outcomes remains unclear. Further studies are needed to clarify the role of *AURKB* rs1059476G>A in cancer.

In the present study, *PTTG1* rs1895320T>C and *RAD21* rs1374297C>G were associated with worse DFS. The *PTTG1* encodes securin, which inhibits the sister-chromatids from separating until late anaphase [29]. Overexpression of *PTTG1* and its relationship with poor prognosis were observed in hepatocellular carcinoma and adrenocortical cancer [27, 30]. Li et al. [31] reported that overexpression of *PTTG1* was associated with lymph node and distant metastases in NSCLC and was correlated with poor OS. RAD21 is a component of the cohesion complex and is involved in chromosome segregation during mitosis, DNA repair, and apoptosis [32]. Enhanced RAD21 expression was associated with early relapse and poor prognosis of breast cancer and *KRAS* mutant colorectal cancer [32, 33]. An association between high expression of *RAD21* and cisplatin resistance was reported in NSCLC [34].

In summary, we found that 4 SNPs (rs7897156, rs1059476, rs1895320, and rs1374297) in mitotic-checkpoint related genes were associated with survival outcomes of NSCLC. In addition, functional data suggest that *BUB3* rs7897156C>T and *AURKB* rs1059476G>A are functional SNPs. Additional studies are required to confirm the effect of these SNPs in diverse ethnic groups.

## MATERIALS AND METHODS

### Study populations

In this study, 766 patients with pathologic stage I, II, and IIIA (micro-invasive N2) NSCLC were enrolled. All patients underwent surgery with curative intent at the Kyungpook National University Hospital, Daegu, Korea (n = 338) or Seoul National University Bundang Hospital, Bundang, Korea (n= 428). Written informed consent was obtain from all patients before surgery. All patients in this study were of Korean ethnicity. This study was approved by the Institutional Review Boards of the Kyungpook National University Hospital and Seoul National University Bundang Hospital.

### SNP selection and genotyping

By searching published review articles, 32 mitotic checkpoint-related genes were selected [8-10, 35, 36]. Using HapMap database, 2035 SNPs in 32 mitotic checkpoint-related genes were collected. Among the 2035 SNPs, 968 SNPs were excluded because the minor allele frequency was < 0.05 in the HapMap JPT data. To identify potentially functional SNPs, the FuncPred in the SNP info web server (http://snpinfo.niehs.nih.gov) was used and 116 potentially functional SNPs were selected from the remaining 1067 SNPs. Among these, 43 SNPs were excluded because of linkage disequilibrium (*r*^2^ > 0.8), based on HaploView (http://broad.mit.edu/mpg/haploview). A total of 73 SNPs in 25 mitotic checkpoint-related genes were genotyped by the MassARRAY® iPLEX assay (SEQUENOM Inc., San Diego, CA, USA). Approximately 5% of the samples were randomly selected to be genotyped again by a different investigator, by a restriction fragment length polymorphism assay, and the results were 100% concordant.

### Promoter-luciferase constructs and luciferase assay

We investigated whether *BUB3* rs7897156C>T modulates the activity of the *BUB3* gene promoter in a luciferase assay. Promoter fragments of *BUB3*, including rs7897156, were synthesized by PCR using the following primers: *BUB3* forward, 5′-GGGGTACCCCGTAGTTTCCACGCGTCCAGC-3′; *BUB3* reverse, 5′-CCGCTCGAGCGG GAAACGGGATCCCCTGCGAA-3′. The PCR products were cloned into the *KpnI/XhoI* site of the pGL3-basic plasmid (Promega, Madison, WI, USA). The correct sequences of all clones were verified by DNA sequencing. The NSCLC cell lines H1299 and A549 were transfected with each reported construct and pRL-SV40 vector (Promega), using Effectene transfection reagent (Qiagen, Hilden, Germany). The cells were collected 48 hour after transfection. Luciferase activity was measured using the Dual-Luciferase® Reporter Assay System (Promega) and the results were normalized based on the activity of Renilla luciferase. All experiments were performed in triplicate.

### RNA preparation and quantitative reverse transcription-PCR (qRT-PCR)

*BUB3* mRNA expression was examined by qRT-PCR. Total RNAs from tumors and paired non-malignant lung tissues (n = 114) were isolated using Trizol (Invitrogen, Carlsbad, USA). Real-time PCR was performed using a LightCycler 480 (Roche Applied Science, Mannheim, Germany) with QuantiFast SYBR Green PCR Master Mix (Qiagen). The real-time PCR primers for *BUB3* and *β-actin* genes involved the following primers: *BUB3* forward, 5′-AGTGTTGGTGTGGGACTTAC-3′; *BUB3* reverse, 5′-AATACTCAACTGCCACTCGG-3′; *β-actin* forward, 5′-TTGTTACAGGAAGTCCCTTGCC-3′; *β-actin* reverse, 5′- ATGCTATCACCTCCCCTGTGT-3′. Each sample was run in duplicate. Relative *BUB3* mRNA expression was normalized to that of *β-actin* expression and then evaluated using the 2^- ΔΔ*C*t^ method [37].

### Construction of three-dimensional model for Aurora kinase

To evaluate the potential effect of *AURKB* rs1059476G>A, which substitutes threonine with methionine at codon 298, the three-dimensional model of the Aurora kinase (PDB ID: 4AF3) SNP variant type was obtained by *in silico* substitution of Thr298 by Met without altering the backbone geometry, using Pymol program (http://pymol.org). The surface electrostatic potential was calculated using DelPhi calculation and displayed using Pymol.

### Construction of expression plasmids for AURKB wild (T298) and variant-type (M298)

To examine the potential effect of wild-type (T298) and variant-type (M298) of AURKB, we constructed wild-type and variant-type plasmids. The AURKB (Myc-DDK-tagged) Human cDNA ORF Clone from Origene Tech (Rockville, MD, USA) was used as a wild-type (T298). The variant-type (M298) construct was created from pCMV6-AURKB-wild-type (T298) using the Quick-change II Site-Directed Mutagenesis kit (Stratagene, Cedar Creek, TX, USA) according to the manufacturer’s protocol. The mutagenesis experiment was amplified using 5’- CCC GCT TCC GTG CCC ATG GGA GCC CAG GAC CTC -3’ (forward) and 5’- GAG GTC CTG GGC TCC CAT GGG CAC GGA AGC GGG -3’ (reverse) primers. Both constructs were verified by sequencing analysis.

### Cell culture and transfection

The 293T cells were cultured in Dulbecco’s modified Eagle’s medium supplemented with 10% heat-inactivated fetal bovine serum. Cells were seeded in a six well plate as the cells were approximately 80∼90% confluent. At the next day, cells were transfected with pCMV6-AURKB-wild-type (T298), pCMV6-AURKB-variant-type (M298), or pCMV6 empty vector using the Lipofectamine 2000 (Invitrogen, Carlsbad, CA, USA) according to the manufacturer’s protocol. After two days from transfection, cells were harvested.

### Western blot

Cell proteins were extracted using M-PER Mammalian Protein Extraction Reagent (Thermo Fisher Scientific, Waltham, MA, USA), supplemented with protease inhibitor and phosphatase inhibitor (Sigma, St. Louis, MO, USA). Western blots were prepared by standard procedures using anti-DDK (Origene Tech, Rockville, MD, USA), anti-actin (Santa Cruz, CA, USA), anti-AKT, anti-phospho-AKT, anti-mTOR, and anti-phospho-mTOR (Cell signaling Technology, Danvers, MA, USA). Immunoreactivity was detected by an Immunobilon Western Chemiluminescent HRP Substrate (Millipore, Billerica, MA, USA).

### Statistical analysis

OS was measured from the day of surgery to the day of death or last follow-up. DFS was counted between the day of surgery and recurrence or death from any cause. The Kaplan-Meier test was performed to assess OS and DFS according to genotype. The aHR and 95% CI were calculated using multivariate Cox proportional hazard models, adjusted for age (< 65 years *vs.* ≥ 65 years), gender (female *vs.* male), smoking status (never *vs.* ever), histological type (squamous cell carcinoma *vs.* adenocarcinoma), pathological stage (stage I *vs.* stage II or IIIA), and adjuvant therapy (yes *vs.* no). A *P* value of less than 0.05 was considered statistically significant. The statistical data were obtained using Statistical Analysis System for Windows, version 9.2 (SAS Institute, Cary, NC, USA).

## SUPPLEMENTARY MATERIALS TABLES






