# Supplementary material for: Polymorphisms in mitotic checkpoint-related genes can influence survival outcomes of early-stage non-small cell lung cancer
Source: Oncotarget. 2017 Jun 27;8(37):61777–85. doi: 10.18632/oncotarget.18693 (PMC5617463; doi:10.18632/oncotarget.18693)
Supplement: Supplementary file 1 [file oncotarget-08-61777-s001.pdf]

Polymorphisms in mitotic checkpoint-related genes can influence survival outcomes of early-stage non-small cell lung cancer

SUPPLEMENTARY MATERIALS

Supplementary Table 1: Summary of polymorphisms of mitotic checkpoint-related genes and survival outcomes in patients with non-small cell lung cancer.

See Supplementary File 1

Supplementary Table 2: The association between polymorphisms and EGFR, ALK, and RET status.

See Supplementary File 2
