# Supplementary material for: Polymorphisms in mitotic checkpoint-related genes can influence survival outcomes of early-stage non-small cell lung cancer
Source: Oncotarget. 2017 Jun 27;8(37):61777–85. doi: 10.18632/oncotarget.18693 (PMC5617463; doi:10.18632/oncotarget.18693)
Supplement: Supplementary file 2 [file oncotarget-08-61777-s002.docx]

| Supplementary Table 1. Summary of polymorphisms of mitotic checkpoint-related genes and survival outcomes in patients with non-small cell lung cancer. | | | | | | | | | | | | | | |
| --- | --- | --- | --- | --- | --- | --- | --- | --- | --- | --- | --- | --- | --- | --- |
| Polymorphism^*^ | | |  | Genotype | |  | *P*^a^ for overall survival | | |  | *P*^a^ for disease free survival | | |  |
| Gene | ID No. | Base change |  | MAF | HWE *P* |  | Dominant | Recessive | Additive |  | Dominant | Recessive | Additive |  |
| *APC* | rs2020383 | T>C |  | 0.18 | 0.55 |  | 0.62 | 0.46 | 0.50 |  | 0.19 | 0.07 | 0.09 |  |
| *APC* | rs414098 | A>G |  | 0.92 | 0.26 |  | 0.70 | 0.41 | 0.99 |  | 0.81 | 0.33 | 0.56 |  |
| *APC* | rs2289485 | T>G |  | 0.92 | 0.06 |  | 0.97 | 0.23 | 0.81 |  | 0.83 | 0.68 | 0.91 |  |
| *APC* | rs459552 | T>A |  | 0.09 | 0.08 |  | 0.36 | - | 0.36 |  | 0.44 | - | 0.44 |  |
| *AURKA* | rs911162 | G>A |  | 0.66 | 0.11 |  | 0.54 | 0.98 | 0.83 |  | 0.07 | 0.40 | 0.15 |  |
| *AURKA* | rs1468056 | G>C |  | 0.47 | 0.13 |  | 0.86 | 0.98 | 0.84 |  | 0.23 | 0.85 | 0.29 |  |
| *AURKA* | rs2064863 | A>C |  | 0.85 | 0.20 |  | 0.63 | 0.62 | 0.56 |  | 0.21 | 0.97 | 0.30 |  |
| *AURKA* | rs2273535 | T>A |  | 0.93 | 0.36 |  | 0.08 | 0.70 | 0.14 |  | 0.71 | 0.39 | 0.48 |  |
| *AURKA* | rs8173 | G>C |  | 0.30 | 0.38 |  | 0.83 | 0.42 | 0.59 |  | 0.13 | 0.79 | 0.32 |  |
| *AURKA* | rs6099128 | T>G |  | 0.96 | 0.15 |  | 0.14 | 0.53 | 0.13 |  | 0.52 | 0.72 | 0.63 |  |
| *AURKA* | rs6127737 | A>G |  | 0.93 | 0.35 |  | 0.14 | 0.50 | 0.16 |  | 0.64 | 0.33 | 0.41 |  |
| *AURKB* | rs1059476 | G>A |  | 0.41 | 0.25 |  | 0.27 | 0.05 | 0.07 |  | 0.15 | 0.11 | 0.06 |  |
| *AURKB* | rs3027260 | G>A |  | 0.28 | 0.48 |  | 0.20 | 0.17 | 0.11 |  | 0.78 | 0.43 | 0.58 |  |
| *BUB1B* | rs2277560 | G>A |  | 0.06 | 0.25 |  | 0.63 | 0.56 | 0.82 |  | 0.91 | 0.77 | 0.99 |  |
| *BUB1B* | rs1801376 | G>A |  | 0.13 | 0.32 |  | 0.50 | 0.54 | 0.42 |  | 0.55 | 0.65 | 0.78 |  |
| *BUB3* | rs7897156 | C>T |  | 0.34 | 0.97 |  | 0.36 | 0.02 | 0.08 |  | 0.90 | 0.17 | 0.47 |  |
| *CDC27* | rs858678 | G>C |  | 0.10 | 0.28 |  | 0.50 | 0.61 | 0.65 |  | 0.71 | 0.12 | 0.95 |  |
| *CDC27* | rs1634265 | A>G |  | 0.24 | 0.16 |  | 0.87 | 0.77 | 0.81 |  | 0.58 | 0.44 | 0.47 |  |
| *CDC27* | rs1102461 | G>C |  | 0.05 | 0.35 |  | 0.25 | 0.43 | 0.61 |  | 0.20 | 0.89 | 0.35 |  |
| *CDK1* | rs1871446 | C>T |  | 0.93 | 0.17 |  | 0.88 | 0.25 | 0.59 |  | 0.83 | 0.42 | 0.92 |  |
| *CENPE* | rs1381657 | C>G |  | 0.10 | 0.37 |  | 0.16 | 0.89 | 0.21 |  | 0.22 | 0.93 | 0.28 |  |
| *CHFR* | rs2306541 | G>A |  | 0.71 | 0.23 |  | 0.78 | 0.98 | 0.80 |  | 0.98 | 0.96 | 0.97 |  |
| *CHFR* | rs3741490 | C>T |  | 0.96 | 0.22 |  | 0.79 | 0.71 | 0.93 |  | 0.96 | 0.72 | 0.92 |  |
| *CHFR* | rs3741489 | T>C |  | 0.87 | 0.47 |  | 0.88 | 0.87 | 0.85 |  | 0.49 | 0.96 | 0.63 |  |
| *ESPL1* | rs1318648 | T>G |  | 0.20 | 0.49 |  | 0.29 | 0.18 | 0.18 |  | 0.06 | 0.82 | 0.10 |  |
| *ESPL1* | rs17125266 | G>A |  | 0.19 | 0.43 |  | 0.24 | 0.25 | 0.16 |  | 0.06 | 0.87 | 0.09 |  |
| *INCENP* | rs7129085 | G>T |  | 0.31 | 0.05 |  | 0.79 | - | 0.79 |  | 0.28 | - | 0.28 |  |
| *KIF11* | rs4933734 | T>A |  | 0.05 | 0.47 |  | 0.32 | 0.53 | 0.39 |  | 0.90 | 0.94 | 0.89 |  |
| *KIF11* | rs7078243 | C>A |  | 0.86 | 0.24 |  | 0.74 | 0.15 | 0.39 |  | 0.49 | 0.47 | 0.40 |  |
| *MAD1L1* | rs12666769 | C>G |  | 0.15 | 0.72 |  | 0.36 | 0.73 | 0.36 |  | 0.66 | 0.27 | 0.47 |  |
| *MAD1L1* | rs11767177 | G>T |  | 0.48 | 0.23 |  | 0.89 | 0.86 | 0.85 |  | 0.95 | 0.67 | 0.91 |  |
| *MAD1L1* | rs3889797 | A>C |  | 0.41 | 0.50 |  | 0.49 | 0.81 | 0.56 |  | 0.69 | 0.81 | 0.69 |  |
| *MAD1L1* | rs4721098 | C>G |  | 0.31 | 0.05 |  | 0.76 | - | 0.76 |  | 0.78 | - | 0.78 |  |
| *MAD2L1* | rs2934379 | A>G |  | 0.10 | 0.17 |  | 0.78 | 0.41 | 0.94 |  | 0.55 | 0.55 | 0.77 |  |
| *MAD2L1* | rs1546120 | C>T |  | 0.11 | 0.08 |  | 0.96 | - | 0.96 |  | 0.50 | - | 0.50 |  |
| *NDC80* | rs2677895 | C>T |  | 0.07 | 0.23 |  | 0.81 | 0.28 | 0.79 |  | 1.00 | 0.08 | 0.44 |  |
| *NDC80* | rs4798003 | G>C |  | 0.60 | 0.27 |  | 0.84 | 0.64 | 0.98 |  | 0.96 | 0.17 | 0.54 |  |
| *NDC80* | rs9051 | G>C |  | 0.05 | 0.12 |  | 0.98 | 0.10 | 0.51 |  | 0.38 | 0.09 | 0.98 |  |
| *NUF2* | rs2292275 | C>T |  | 0.08 | 0.39 |  | 0.14 | 0.31 | 0.13 |  | 0.70 | 0.49 | 0.54 |  |
| *NUF2* | rs2292274 | T>C |  | 0.88 | 0.23 |  | 0.74 | 0.75 | 0.87 |  | 0.70 | 0.84 | 0.81 |  |
| *PLK1* | rs27770 | T>C |  | 0.67 | 0.47 |  | 0.52 | 0.55 | 0.97 |  | 0.61 | 0.42 | 0.87 |  |
| *PLK3* | rs17881698 | A>G |  | 0.47 | 0.26 |  | 0.50 | 0.85 | 0.53 |  | 0.64 | 0.81 | 0.77 |  |
| *PTTG1* | rs1895320 | T>C |  | 0.16 | 0.63 |  | 0.16 | 0.14 | 0.44 |  | 0.45 | 0.001 | 0.11 |  |
| *PTTG1* | rs2910200 | C>T |  | 0.98 | 0.13 |  | 0.51 | 0.79 | 0.51 |  | 0.83 | 0.42 | 0.67 |  |
| *PTTG1* | rs2910201 | C>T |  | 0.84 | 0.29 |  | 0.92 | 0.89 | 0.98 |  | 0.90 | 0.98 | 0.93 |  |
| *RAD21* | rs1348798 | T>A |  | 0.15 | 0.44 |  | 0.76 | 0.74 | 0.86 |  | 0.80 | 0.71 | 0.73 |  |
| *RAD21* | rs1374297 | C>G |  | 0.41 | 0.80 |  | 0.48 | 0.28 | 0.29 |  | 0.16 | 0.05 | 0.04 |  |
| *RAD21* | rs6987652 | G>A |  | 0.52 | 0.15 |  | 0.06 | 0.55 | 0.06 |  | 0.26 | 0.68 | 0.26 |  |
| *RAD21* | rs10107209 | C>T |  | 0.34 | 0.15 |  | 0.09 | 0.63 | 0.09 |  | 0.37 | 0.81 | 0.38 |  |
| *RAD21* | rs10110234 | C>T |  | 0.25 | 0.15 |  | 0.11 | 0.84 | 0.15 |  | 0.33 | 0.67 | 0.42 |  |
| *RNF2* | rs1046592 | A>G |  | 0.06 | 0.38 |  | 0.37 | 0.06 | 0.10 |  | 0.95 | 0.31 | 0.57 |  |
| *RNF2* | rs3766723 | T>G |  | 0.06 | 0.31 |  | 0.32 | 0.60 | 0.33 |  | 0.67 | 0.87 | 0.70 |  |
| *SGOL2* | rs1036533 | C>T |  | 0.22 | 0.10 |  | 0.54 | 0.62 | 0.49 |  | 0.91 | 0.62 | 0.80 |  |
| *SGOL2* | rs999576 | T>C |  | 0.19 | 0.27 |  | 0.74 | 0.98 | 0.81 |  | 0.91 | 0.51 | 0.84 |  |
| *TTK* | rs240228 | G>A |  | 0.51 | 0.29 |  | 0.75 | 0.64 | 0.94 |  | 0.52 | 0.76 | 0.52 |  |
| *ZWILCH* | rs3087660 | A>G |  | 0.49 | 0.44 |  | 0.99 | 0.07 | 0.27 |  | 0.65 | 0.57 | 0.95 |  |
| *ZWILCH* | rs4776787 | G>C |  | 0.61 | 0.29 |  | 0.16 | 0.76 | 0.22 |  | 0.64 | 0.95 | 0.69 |  |
| *ZWILCH* | rs11071896 | A>G |  | 0.99 | 0.12 |  | 0.48 | 0.94 | 0.54 |  | 0.60 | 0.52 | 0.52 |  |
| MAF, minor allele frequency; HWE *P*, *P* for Hardy-Weinberg equilibrium test.  ^*^ Fourteen polymorphisms in 12 genes were excluded; 6 of MAF < 0.05 in the study population, 7 of HWE *P* < 0.05, and 1 of call rate < 95%.  ^a^ *P*-values were calculated using multivariate Cox proportional hazard models, adjusted for age, sex, smoking status, tumor histology, pathologic stage, and adjuvant therapy. | | | | | | | | | | | | | | |
