# Supplementary material for: Polymorphisms in mitotic checkpoint-related genes can influence survival outcomes of early-stage non-small cell lung cancer
Source: Oncotarget. 2017 Jun 27;8(37):61777–85. doi: 10.18632/oncotarget.18693 (PMC5617463; doi:10.18632/oncotarget.18693)
Supplement: Supplementary file 3 [file oncotarget-08-61777-s003.docx]

| Supplementary Table 2. The association between polymorphisms and EGFR, ALK, and RET status. | | | | | | | | | | | | | | | |
| --- | --- | --- | --- | --- | --- | --- | --- | --- | --- | --- | --- | --- | --- | --- | --- |
| Cell type | Polymorphism | EGFR |  |  |  |  | ALK |  |  |  |  | RET |  |  |  |
|  | Genotype | negative | positive | unknown | *P* |  | negative | positive | unknown | *P* |  | negative | positive | unknown | *P* |
| All | rs7897156 |  |  |  |  |  |  |  |  |  |  |  |  |  |  |
|  | CC | 97 | 88 | 140 | 0.95 |  | 31 | 0 | 293 | 0.32 |  | 31 | 0 | 293 | 0.73 |
|  | CT | 98 | 95 | 146 |  |  | 25 | 3 | 311 |  |  | 26 | 1 | 312 |  |
|  | TT | 27 | 21 | 41 |  |  | 7 | 0 | 82 |  |  | 7 | 0 | 82 |  |
|  | rs1059476 |  |  |  |  |  |  |  |  |  |  |  |  |  |  |
|  | GG | 78 | 66 | 115 | 0.86 |  | 25 | 2 | 231 | 0.44 |  | 26 | 0 | 232 | 0.49 |
|  | GA | 112 | 109 | 157 |  |  | 33 | 1 | 344 |  |  | 33 | 1 | 344 |  |
|  | AA | 32 | 31 | 53 |  |  | 6 | 0 | 110 |  |  | 6 | 0 | 110 |  |
|  | rs1895320 |  |  |  |  |  |  |  |  |  |  |  |  |  |  |
|  | TT | 152 | 147 | 221 | 0.23 |  | 47 | 2 | 470 | 0.74 |  | 47 | 1 | 471 | 0.69 |
|  | TC | 66 | 49 | 92 |  |  | 17 | 1 | 189 |  |  | 18 | 0 | 189 |  |
|  | CC | 3 | 3 | 12 |  |  | 0 | 0 | 18 |  |  | 0 | 0 | 18 |  |
|  | rs1374297 |  |  |  |  |  |  |  |  |  |  |  |  |  |  |
|  | CC | 71 | 66 | 123 | 0.06 |  | 19 | 0 | 240 | 0.44 |  | 19 | 0 | 240 | 0.47 |
|  | CG | 110 | 111 | 137 |  |  | 32 | 2 | 321 |  |  | 36 | 1 | 321 |  |
|  | GG | 40 | 26 | 62 |  |  | 8 | 1 | 119 |  |  | 8 | 0 | 120 |  |
|  |  |  |  |  |  |  |  |  |  |  |  |  |  |  |  |
| AC | rs7897156 |  |  |  |  |  |  |  |  |  |  |  |  |  |  |
|  | CC | 66 | 70 | 49 | 0.65 |  | 21 | 0 | 163 | 0.30 |  | 21 | 0 | 163 | 0.73 |
|  | CT | 71 | 62 | 40 |  |  | 16 | 3 | 154 |  |  | 17 | 1 | 155 |  |
|  | TT | 17 | 12 | 13 |  |  | 6 | 0 | 36 |  |  | 6 | 0 | 36 |  |
|  | rs1059476 |  |  |  |  |  |  |  |  |  |  |  |  |  |  |
|  | GG | 53 | 47 | 33 | 0.72 |  | 16 | 2 | 114 | 0.75 |  | 17 | 0 | 115 | 0.85 |
|  | GA | 83 | 71 | 54 |  |  | 22 | 1 | 185 |  |  | 22 | 1 | 185 |  |
|  | AA | 18 | 25 | 15 |  |  | 9 | 0 | 52 |  |  | 6 | 0 | 52 |  |
|  | rs1895320 |  |  |  |  |  |  |  |  |  |  |  |  |  |  |
|  | TT | 110 | 108 | 73 | 0.15 |  | 32 | 2 | 256 | 0.86 |  | 32 | 1 | 257 | 0.75 |
|  | TC | 43 | 31 | 24 |  |  | 13 | 1 | 84 |  |  | 14 | 0 | 84 |  |
|  | CC | 1 | 1 | 4 |  |  | 0 | 0 | 6 |  |  | 0 | 0 | 6 |  |
|  | rs1374297 |  |  |  |  |  |  |  |  |  |  |  |  |  |  |
|  | CC | 44 | 47 | 40 | 0.23 |  | 10 | 0 | 120 | 0.33 |  | 10 | 0 | 120 | 0.35 |
|  | CG | 82 | 75 | 41 |  |  | 27 | 2 | 167 |  |  | 28 | 1 | 169 |  |
|  | GG | 29 | 21 | 20 |  |  | 7 | 1 | 62 |  |  | 7 | 0 | 63 |  |
| AC, adenocarcinoma; EGFR, epidermal growth factor receptor; ALK, anaplastic lymphoma kinase; RET, ret proto-oncogene | | | | | | | | | | | | | | | |
